# Evaluation of the theory-based Quality Improvement in Physical Therapy (QUIP) programme: a one-group, pre-test post-test pilot study

**DOI:** 10.1186/1472-6963-13-194

**Published:** 2013-05-25

**Authors:** Geert M Rutten, Janneke Harting, L Kay Bartholomew, Angelique Schlief, Rob AB Oostendorp, Nanne K de Vries

**Affiliations:** 1 Department of Health Promotion, Faculty of Health, Medicine and Life Sciences, Maastricht University, Maastricht, the Netherlands; 2 Department of Public Health, Academic Medical Centre University of Amsterdam, Amsterdam, The Netherlands; 3 Division of Health Promotion and Behavioral Sciences, School of Public Health, University of Texas, Houston, TX, USA; 4 Scientific Institute for Quality in Healthcare, Radboud University Nijmegen Medical Centre, Nijmegen, The Netherlands

**Keywords:** Guideline implementation, Quality improvement, Multilevel programme, Individual professional, Practice management, Physical therapy

## Abstract

**Background:**

Guideline adherence in physical therapy is far from optimal, which has consequences for the effectiveness and efficiency of physical therapy care. Programmes to enhance guideline adherence have, so far, been relatively ineffective. We systematically developed a theory-based Quality Improvement in Physical Therapy (QUIP) programme aimed at the individual performance level (practicing physiotherapists; PTs) and the practice organization level (practice quality manager; PQM). The aim of the study was to pilot test the multilevel QUIP programme’s effectiveness and the fidelity, acceptability and feasibility of its implementation.

**Methods:**

A one-group, pre-test, post-test pilot study (N = 8 practices; N = 32 PTs, 8 of whom were also PQMs) done between September and December 2009. Guideline adherence was measured using clinical vignettes that addressed 12 quality indicators reflecting the guidelines’ main recommendations. Determinants of adherence were measured using quantitative methods (questionnaires). Delivery of the programme and management changes were assessed using qualitative methods (observations, group interviews, and document analyses). Changes in adherence and determinants were tested in the paired samples T-tests and expressed in effect sizes (Cohen’s d).

**Results:**

Overall adherence did not change (3.1%; p = .138). Adherence to three quality indicators improved (8%, 24%, 43%; .000 ≤ p ≤ .023). Adherence to one quality indicator decreased (−15.7%; p = .004). Scores on various determinants of individual performance improved and favourable changes at practice organizational level were observed. Improvements were associated with the programme’s multilevel approach, collective goal setting, and the application of self-regulation; unfavourable findings with programme deficits. The one-group pre-test post-test design limits the internal validity of the study, the self-selected sample its external validity.

**Conclusions:**

The QUIP programme has the potential to change physical therapy practice but needs considerable revision to induce the ongoing quality improvement process that is required to optimize overall guideline adherence. To assess its value, the programme needs to be tested in a randomized controlled trial.

## Background

Although clinical guidelines are seen as a bridge between evidence and practice [1,2], their uptake in routine practical performance has been limited [3,4]. This incomplete implementation is attributed to factors related to individual professionals [5,6], organizational issues [7,8], patients [9,10], and guideline quality [11]. Programmes to enhance guideline adherence have had limited effect. An extensive 2004 review of implementation interventions found 5–15% improved application of evidence-based practices, but multifaceted interventions performed no better than single-focus interventions [4]. These findings were comparable to adherence improvement found in a systematic review in allied health care literature [12] and, except for some self-reported improvements [13], with the effectiveness of physical therapy guideline implementation interventions [14,15].

There are several reasons proposed for the modest effectiveness of implementation interventions. First, the underlying problem may be poorly described. Often problem analysis in implementation research depends on either qualitative or quantitative methods, where a combination is recommended [16]. A second reason is the limited application of theoretical frameworks in implementation research, including problems with selecting applicable theoretical constructs relevant to the implementation intervention’s design [4,17]. Together, this suggests a lack of a rationale for the mechanism of change in an intervention [4,18]. Finally, there has been a narrow intervention focus on individual professionals instead of combining it with efforts to change organizational or broader environmental and cultural factors [19-21]. Taking these issues into consideration, we systematically developed a theory-based intervention. The goal of this Quality Improvement in Physical Therapy (QUIP) programme was to improve physical therapists’ adherence to the Dutch guidelines for low back pain [22,23], since this was the most prevalent diagnosis in Dutch private practice physical therapy [24]. In addition, adherence of Dutch physical therapists to these guidelines had repeatedly been shown to be limited (42%-67%) [14,25,26], while previous studies, including a study on the Dutch guidelines for low back pain, indicated that higher adherence rates were related to better treatment results and lower utilization of care [26,27].

The guidelines urge clinical reasoning, assessment and management of psychosocial factors and documentation including outcome measurement. Their four main features are: applying the International Classification of Functioning, Disability and Health (ICF); identifying and applying patient profiles with duration, course, and psychosocial factors influencing recovery; limiting the number of treatment sessions in case of acute low back pain; and focusing on patient behaviour to restore physical activity and social participation (an English version is available at http://www.fysionet-evidencebased.nl/index.php/kngf-guidelines-in-english). At the time of the study, the Dutch physical therapy guidelines were disseminated by a combination of strategies. These included sending them by mail to every member of the Royal Dutch Society for Physical Therapy (KNGF), which are about 90% of Dutch physical therapists, presentation at the annual national physical therapy conference and publication in the National Journal of Physical Therapy. The guidelines also came with a competency manual in which physical therapists could test their knowledge of the guideline. At a later stage the Society for Physical Therapy developed programmes that Communities of Practice existing in Dutch physical therapy could use to improve guideline implementation. All implementation activities were voluntary and targeting individual physical therapists only.

In our preparatory problem analysis, we combined the Precede stages of the Precede-Proceed model [28] with Rogers’ Diffusion of Innovations theory [29] to qualitatively and quantitatively assess motivational, affective and organizational determinants of guideline adherence [30,31]. The results suggested that determinants at five levels contributed to lack of guideline adherence: individual professional, practice management, professional organization, patient and guidelines. For physical therapists (PTs), important subjects to improve were clinical reasoning, applying the categories of the International Classification of Functioning, Disability and Health (ICF) [32], managing psychosocial factors, maintaining complete patient records, and using measurement instruments (health outcome questionnaires). It was important for quality managers of the practice (PQMs) to improve monitoring of both organizational aspects of the practice and the performance of the individual PTs. This would require changes in practice structure and culture, including holding deliberation meetings, assuring availability of materials and resources (personal and material) for quality improvement, effectively using an electronic patient record, formulating collective quality objectives, and creating or maintaining an atmosphere of openness and respect. With regard to low back pain guidelines, issues to improve were comprehensiveness, user friendliness and ability to support clinical reasoning.

To develop the QUIP programme, we applied Intervention Mapping (IM) [33], a systematic approach to link theoretical methods and their practical applications of change to influential determinants. We focused on three levels: individual physical therapists (PTs), practice quality managers (PQMs), and the Dutch guidelines for low back pain, which were under revision at that time. This paper describes the pilot study of the QUIP programme. In IM, a pilot study informs the further development of the programme towards a final version. For this purpose two questions are important: 1) is it likely that the programme is effective, in other words, is further development worthwhile, and 2) is the present format of the programme feasible, which is only of importance if the answer to the first question is affirmative. In accordance, this study comprised an evaluation of the potential effectiveness of the programme as regards the improvement of guideline adherence and its determinants, and a process evaluation, to evaluate the programme’s implementation feasibility, acceptability and fidelity. The combination of the effect and the process evaluation findings additionally informed the identification of strengths and weaknesses of the programme [34].

## Methods

### Intervention

**Figure 1:**
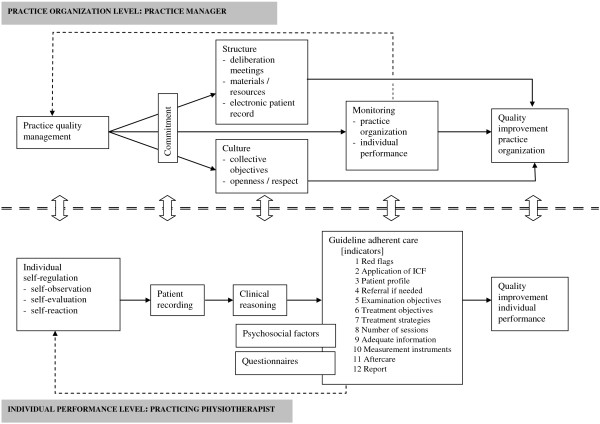
Programme theory of the Quality Improvement in Physical Therapy (QUIP) programme.

The QUIP programme [35] had three objectives (Figure 1 Programme theory): to teach PTs a method to improve or maintain their quality of care, to give PQMs tools to accomplish quality management, and to make PTs and PQMs aware that quality of care requires team effort and to help them achieve it. A synopsis of the QUIP programme is presented in Additional file 1 (for a detailed description, see Additional file 2).

### Design and recruitment

Since this pilot study informed the further development of the programme, the potential effects of the QUIP programme were evaluated in a single group, pre-test post-test design. This design is not appropriate to thoroughly assess the effectiveness of an intervention, but it is productive when pre-test data are obtained shortly before and post-test data shortly after the intervention [36]. The process evaluation was an observational study guided by questions (Table 1) based on the programme theory. We used purposive sampling. This approach intends to include participants that are expected to be informative for the study purpose, which is especially useful for pilot studies of newly developed interventions [36]. Inclusion criteria required that the practices have an initial quality management structure with a (starting) PQM and at least five PTs. In order to reach an acceptable reflection of common practice, we preferred a mixture of male and female therapists of various age groups and with a difference in working experience. We also intended to include PTs and manipulative PTs (MPTs), since a substantial proportion of low back pain patients visit MPTs. For pragmatic reasons, such as the opportunity for sufficient interaction and attention for individual participants during the programme, we intended to include no more than 8 practices, including 40 PTs 8 of whom would also be PQMs. For logistic convenience, the practices were predominantly located in the southern part of the Netherlands.

**Table 1:** Overview of the topics, questions and methods of the effect and process evaluation

**Measurement instrument (completed by)**	**Clinical vignettes with quality indicators**	**Self report questionnaire**	**Observations with coding sheet**	**Group interviews**	**Document analysis of PDP’s and PQIP’s**	**Field notes**	**General evaluation questionnaire**
	**(Participants)**	**(Participants)**	**(GR, JH)**	**(GR, JH, AS)**	**(Participants)**	**(GR, JH, AS)**	**(Participants)**
**Guideline adherence**	**✓**						
**Individual and organizational determinants**		**✓**		**✓**		**✓**	
**Fidelity**							
**Content of the intervention**							
1. Which important subjects from the analyses are addressed in the intervention?			**✓**	**✓**	**✓**		
2. Which methods and applications are actually applied (PT and PQM)?			**✓**	**✓**			
3. Which determinants are addressed during the intervention?			**✓**	**✓**			
**Execution of the intervention**							
4. Are methods and applications applied as intended (why not)?			**✓**	**✓**			
5. How is the extent of participation to the individual modules of the intervention?			**✓**				
**Acceptability**							
**Materials**							
6. How do participants judge the concept of the revised guideline?			**✓**	**✓**			
7. Do PTs apply the patient information leaflet and why/why not?				**✓**	**✓**	**✓**	
**General**							
8. Do the participants evaluate the intervention as acceptable (tailored to personal level; sufficient interaction; providing new and useful knowledge and skills)?			**✓**	**✓**	**✓**		**✓**
9. How do the participants value the intervention and its individual applications?			**✓**	**✓**			**✓**
10. How do the participants value the trainers of the intervention?				**✓**	**✓**		**✓**
11. Does the intervention evoke higher commitment to quality management (PQP, monitoring of this PQP, sustaining the quality management)?				**✓**	**✓**	**✓**	
**Feasibility**							
12. Is the implementation of the intervention in its current form feasible?			**✓**	**✓**		**✓**	
13. If not, what should change to enhance the feasibility of the programme?			**✓**	**✓**			

In spring 2009, we approached two national private practice networks that encompass 150 practices and that demand a quality certification from their members. Quality certification is more and more the standard in Dutch private practice physical therapy. The network managers contacted all practices by email that announced the study and explained its purpose as well as the conditions for participation. The email message included a registration form. Fifteen practices showed interest. The researchers telephoned practices to invite them to participate and to further explain the study purpose and requirements for participation. Based on these phone calls, 8 practices were willing and eligible to participate. All the participants signed for informed consent before they enrolled in the study.

### Measurements and data collection

#### Effect evaluation

*Guideline adherence* of individual PTs was measured with four clinical vignettes (see Table 1). The clinical vignettes were developed in an iterative process with an expert team, and were pretested before use. They were based on validated vignettes from a previous study, which showed to have acceptable validity (Spearman’s *r*_*s*_ = .31) to measure PTs’ guideline adherence [37-39]. The vignettes covered 12 quality indicators based on guideline recommendations [40] and represented patients with non-specific low back pain and a favourable natural course, a delayed course without psychosocial factors, and a delayed course with psychosocial factors and a patient with specific low back pain. The scores on the individual quality indicators (1 = meets quality indicator, 0 = does not meet quality indicator) per vignette were used to calculate an overall percentage score per indicator per therapist: (actual score on indicator/maximum score on indicator) × 100. The mean overall percentage adherence was established by calculating the average score of the 12 indicators.

*Clinical reasoning* was measured by assessing the consistency of PTs’ choices over three separate quality indicators (see bottom part of Table 2) concerning the handling of psychosocial factors [26]. Consistency in choices was operationalised as the presence (1 = present, 0 = not present) of the “conditional argument” (if-then connective) which is an important component of human reasoning [41]. For instance, if PTs identified psychosocial factors in the case description of a vignette, did they subsequently address them in their treatment objectives? In accordance with the overall adherence score, the overall consistency measure was determined by calculating the average of the three consistency indicator scores.

**Table 2:** Changes in percentage of adherence after the Quality Improvement in Physical Therapy programme

	**Pretest mean (SD)**	**Post-test mean (SD)**	**t**	**df**	**p**	**Effect size Cohen’s d**
	**(n = 24)**	**(n = 24)**				
**Overall adherence**	51.5 (8.7)	54.6 (9.0)	−1.535	23	.138	0.35^b^
**Quality indicators**						
1. Assessment of red flags	93.5 (11.2)	89.6 (14.6)	1.164	22	.257	- 0.30^a^
2. Application of ICF	5.5 (12.7)	8.3 (14.7)	−0.700	23	.491	0.20^a^
3. Correct patient profile	55.9 (19.2)	40.2 (19.6)	3.296	19	.004**	- 0.81^c^
4. Referral if needed	95.8 (9.5)	97.9 (7.1)	−0.811	23	.426	0.25^a^
5. Applicable examination objectives	4.2 (11.3)	9.7 (23.0)	−1.072	23	.295	0.30^a^
6. Applicable treatment objectives	38.9 (27.2)	30.6 (29.3)	1.661	23	.110	- 0.29^a^
7. Applicable treatment strategies	30.6 (21.8)	37.5 (22.6)	−1.415	23	.170	0.32^a^
8. Limit number of sessions if course is favourable	41.7 (50.4)	66.7 (48.1)	−2.015	23	.056^#^	0.51^b^
9. Adequate information	11.1 (18.8)	19.4 (27.7)	−1.238	23	.228	0.35^b^
10. Complete evaluation	27.8 (40.1)	44.4 (38.9)	−1.313	23	.202	0.42^b^
10a. Used measurement instruments	38.9 (44.7)	81.9 (21.9)	−4.251	23	.000**	1.22^c^
11. Aftercare arranged	84.7 (26.0)	90.3 (25.0)	−1.072	23	.295	0.22^a^
12. Report to physician	91.7 (28.2)	93.1 (24.0)	−0.272	23	.788	0.05^a^
**Consistency with regards to psychosocial factors**						
Overall consistency in handling influential psychosocial factors	59.7 (16.3)	68.1 (18.0)	−2.432	23	.023**	0.49^b^
a. Choosing examination objectives about psychosocial factors	58.3 (26.5)	56.9 (36.1)	0.189	23	.852	- 0.04^a^
b. Choosing treatment objectives which involve psychosocial factors	33.3 (26.0)	56.9 (31.8)	−3.093	23	.005**	0.81^c^
c. Choosing to provide information about psychosocial factors	87.5 (19.2)	90.3 (15.5)	−0.624	23	.539	0.16^a^

**p < .01, *p < .05, #p < .10.

^a^ = small ES, ^b^ = medium ES, ^c^ = large ES.

Changes in *individual level determinants* (see Table 3) were measured using a self-report questionnaire developed in an earlier stage of the study [31]. For every determinant, the questionnaire contained one or more items using a five-point Likert scale (1 = completely disagree to 5 = completely agree). The individual as well as organizational level determinants resulted from a factor analysis. Cronbach’s α ranged from 0.58 to 0.87 for the different determinant scales.

**Table 3:** Changes in scores on influential determinants of adherence after the Quality Improvement in Physical Therapy programme (1 = disagree to 5 = agree)

	**N**_**items**_	**α**	**Pretest mean (SD)**	**Post-test mean (SD)**	**t**	**df**	**p**	**Effect size Cohen’s d**
			**(n = 25)**	**(n = 25)**				
**Individual level**								
Attention paid to the guideline	2	0.70	3.3 (0.6)	4.0 (0.6)	−4.047	24	.000**	1.17^c^
Compatibility with way of working	4	0.70	3.3 (0.6)	3.5 (0.6)	−1.342	24	.192	0.33^b^
Compatibility with patient demands	3	0.78	3.2 (0.8)	3.6 (0.5)	−3.166	24	.004**	0.60^c^
Flexibility of the guideline	5	0.87	3.5 (0.6)	3.8 (0.5)	−2.120	24	.045*	0.54^b^
Communicability of the guideline	3	0.82	4.0 (0.6)	4.2 (0.5)	−1.454	24	.159	0.36^b^
Visibility of results of the guideline	4	0.89	2.9 (0.8)	3.3 (0.8)	−2.520	24	.019*	0.50^b^
Feeling pride/confidence	6	0.86	3.0 (0.7)	3.6 (0.6)	−4.688	24	.000**	0.92^c^
Feeling uncomfortable	6	0.81	3.0 (0.6)	2.5 (0.5)	3.594	24	.001**	- 0.90^c^
Self-efficacy to apply questionnaires (behavioural SE)	5	0.71	3.5 (0.6)	4.0 (0.5)	−4.804	24	.000**	0.90^c^
Self-efficacy to overcome barriers (tensional SE)	2	0.82	3.0 (0.8)	3.6 (0.8)	−3.343	24	.003**	0.75^c^
Self-efficacy towards perceived social pressure (social SE)	5	0.84	3.6 (0.5)	3.9 (0.5)	−2.031	23	.031*	0.60^c^
Self-efficacy to explain hands off policy to patients	1	--	3.8 (0.7)	4.2 (0.6)	−1.995	24	.058	0.61^c^
Self-efficacy to deal with psychosocial factors	1	--	3.5 (1.0)	3.9 (0.7)	−2.089	24	.047*	0.46^b^
Potential losses	5	0.85	2.0 (0.6)	1.7 (0.7)	1.815	24	.082	- 0.46^b^
Social norm of colleagues	2	0.72	2.8 (0.8)	3.4 (0.8)	−3.055	24	.005**	0.75^c^
Social norm: perceived behaviour of peers	1	--	2.9 (1.2)	3.3 (1.2)	−1.809	24	.083	0.33^b^
Motivation to comply with colleagues	3	0.58	3.1 (0.7)	3.4 (0.7)	−2.413	24	.024**	0.43^b^
Social norm of patient	1	--	2.7 (1.1)	2.8 (0.8)	−0.146	24	.885	0.10^a^
Motivation to comply with patient	1	--	4.1 (0.7)	4.0 (0.8)	1.000	24	.327	- 0.13^a^
Barriers logistic	5	0.81	2.6 (0.7)	2.3 (0.9)	1.394	24	.176	- 0.37^b^
Barriers working part time	1	--	1.5 (0.8)	1.4 (0.7)	0.225	24	.824	- 0.13^a^
Barriers market directed care	1	--	2.2 (1.0)	2.0 (1.1)	1.238	24	.228	- 0.19^a^
Incompatibility other guidelines	2	0.69	2.0 (0.7)	1.8 (0.7)	1.429	24	.166	- 0.29^a^
Feeling uncertain about position	3	0.76	2.4 (0.6)	1.9 (0.6)	2.850	24	.009**	- 0.83^c^
**Practice level**								
Regular deliberative meetings	1	--	3.4 (1.0)	3.9 (0.6)	−2.701	24	.012*	0.61^c^
Practice arrangements about treatment of patients with low back pain	1	--	2.6 (1.4)	3.5 (1.3)	−3.366	24	.003**	0.67^c^
Guideline is part of practices routine	1	--	3.0 (0.9)	3.3 (1.1)	−1.572	24	.129	0.30^a^
Arrangements with other disciplines	2	0.82	2.3 (1.1)	2.6 (1.3)	−1.664	23	.110	0.25^a^
Culture of education/training	1	--	4.3 (0.7)	4.2 (0.7)	0.527	24	.603	- 0.14^a^
Handling measurement instruments	1	--	2.4 (0.9)	3.3 (1.1)	−4.028	24	.000**	0.90^c^
Availability guidelines/instruments	3	0.79	4.5 (0.6)	4.6 (0.5)	−1.372	24	.183	0.20^a^
Supportive practice culture	3	0.65	4.4 (0.5)	4.4 (0.5)	0.249	24	.805	0.0

**p < .01, *p < .05, #p < .10.

^a^ = small ES, ^b^ = medium ES, ^c^ = large ES.

*Organizational level determinants* were partly measured using the questionnaire for individual PTs. Changes in the organization were also assessed with observation, group interviews, field notes and document analyses. In addition to the questionnaire, we focused on self regulation, commitment to quality management, transfer of learned information to the practice, patient recording, presence of regular deliberation meetings, facilitation of questionnaire use, presence of a monitoring structure, and structures for maintenance.

The clinical vignettes as well as the determinants questionnaire were completed by the PTs and the PQMs one week before the start of the intervention (August 2009), and within two weeks after finishing the intervention (December 2009). Completing these measures took 60 to 90 minutes.

#### Process evaluation

Applying the principle of triangulation [36], we used both quantitative and qualitative process evaluation methods (Table 1). During every programme session, two members of the research team (GR and JH) were present for observation. The observers used a coding sheet to check off the change objectives, determinants, and the planned methods and strategies that were addressed. They independently made notes about the quality of delivery of the programme components.

Six participant group or individual interviews (n = 21) were conducted by two members of the research team (GR and JH or AS) within 3 weeks after the programme ended. Two interviews, one after the second session and one after the final session, were conducted with the instructors who executed the programme. Guided by the evaluation questions, one of the researchers performed the interview and the other took notes. Visiting six practice locations for the interviews also provided the researchers the opportunity to observe changes in practice management and to make field notes.

Documents to be evaluated were the Personal Development Plans (n = 25) and the Practice Quality Improvement Plans (n = 7) that were written by the PTs and the PQMs as an assignment of the programme. After the last session, the participants completed a general course evaluation questionnaire (n = 25; completion time less than 15 minutes) to assess perceptions of content quality, trainers, location, organization and overall judgment of the course (1 = extremely bad to 10 = excellent).

### Analysis

#### Effect analysis

For the PTs, descriptive statistics revealed mean scores and changes in pre- and post-intervention means for overall adherence, clinical reasoning indicators by means of choice consistency, perceived adherence and individual level determinants.

For organizational level determinants, we compared pre- and post-intervention means of the organizational items in the questionnaire for the individual PT. We assessed organizational changes by means of qualitative content analysis with an open coding approach [36], finding patterns in the observation, interviews, and field notes.

Due to the small sample size, we combined paired sample t-tests with Cohen’s d to express the changes in adherence and individual and organizational determinants in effect sizes (ES). As recommended for correlated designs [42], Cohen’s d was computed using original means and standard deviations pre- and post-intervention. ESs were categorized as small (≤ 0.32), medium (0.33 - 0.55) or large (≥ 0.56) [43]. Since we aimed to assess the potential effectiveness of our programme we chose a less restrictive level of α = .05.

The statistical software SPSS15 for Windows was used for all quantitative analyses.

#### Process analysis

First, the findings were combined for each method separately. The coding sheets and observational notes were assembled in the week after each programme session. In the week following each interview, all interview notes and practice observations were processed. The PDPs and PQIPs were evaluated for their completeness and the plans’ attainability by judging the capability of PTs and PQMs to set effective individual and collective quality improvement goals and by assessing the presence of an explicit plan for continuing quality improvement activity after the intervention’s end. Descriptive statistics revealed the average participants’ perceptions of the course. Next, qualitative content analysis of all observations, interviews, documents, and field notes was performed, interchangeably using a template and a comparative method of data analysis [36]. The findings searched for patterns of each method and subsequently between methods, and grouped by research question related to fidelity, acceptability and feasibility (Table 1). These summarized findings were discussed until consensus was reached about their interpretation.

### Ethical approval

The study was approved by the Committee on Medical Research Involving Human Subjects (CMO) Arnhem-Nijmegen (Filenr. CMO 2007/172).

## Results

### Response and participants

Thirty-one PTs from 8 practices participated. In every practice, one of the participants was the PQM. One practice (including 4 PTs) decided to withdraw during the programme; 7 practices including 27 PTs completed the course.

The average age of the participants was 39 years (range 24 to 56), 55% were female (n = 15), and participants averaged 15.5 years of work experience (SD = 9.86). Almost 33% (n = 8) were MPTs. On the average, PQMs had 0.35 FTE (range 0.1–0.5) available for quality management.

One participant did not complete the vignettes at the start of the programme, one did not complete the determinant questionnaire and the vignettes after the programme, and one completed the determinant questionnaire too late for data analysis. In the end, 24 vignette pre-test and post-test measurements and 25 determinant questionnaires were available for analysis.

### Effectiveness

#### Adherence

Overall guideline adherence did not change (3%; p = .138, ES = 0.35; Table 2). Three quality indicators improved: the use of measurement instruments (p = .000, ES = 1.22), consistency in choosing treatment objectives involving psychosocial factors (p = .005, ES = 0.81), and overall consistency in handling psychosocial factors (p = .023, ES = 0.49). One quality indicator declined: the choice of the correct patient profile (p = .004, ES = −0.81).

#### Individual level determinants

Improvements were found for various determinants at the individual level (.000 ≤ p ≤ .047, 0.43 ≤ ES ≤ 1.17; Table 3). PTs paid more attention to the guideline and found the guideline more compatible with patient demands. They expressed more pride and confidence and less discomfort when they applied the guideline. Their self-efficacy expectations towards using questionnaires to overcome barriers and to deal with social pressure increased. They had increased positive perceptions about the social norms of their colleagues and felt more certain about their position in treating patients with low back pain. They also perceived the guideline as more flexible and the results of guideline-adherent care as more visible. Their self-efficacy expectations to deal with psychosocial factors increased, and they showed higher motivation to comply with colleagues.

#### Management changes

Three organizational level determinants improved. Practices managers organized more deliberation meetings (p = .012; ES = 0.61), made more practice arrangements about the treatment of patients with low back pain (p = .003; ES = 0.67), and better organized the handling of measurement instruments in their practices (p = .000, ES = 0.90; see Table 3).

Our qualitative analyses revealed that, with respect to changes in management structure, participants indicated that ‘following the programme had put things in motion’. The PQMs’ schedules showed more deliberation meetings to discuss plans for low back pain patients. Participants of practices with lower baseline management levels expressed that the management had moved toward a better-structured process, and PQMs of practices with a higher baseline management level showed the results of their application of the newly learned management tools into their practices. The management scan for organization assessment was used in two practices. Moreover, we observed that practices had organized space and trained staff to help patients to complete questionnaires. As regards involvement of staff in quality management, all participants expressed increased awareness that improving quality of care is a team effort. Employees reported feeling greater involvement in practice policy. Self-regulation was reflected in that PTs could introduce a topic in the meetings and together with the managers could decide whether a quality improvement activity was necessary.

For sustainability individual practices introduced other organizational and cultural management innovations. Some showed how they implemented a buddy system in which two colleagues checked each other’s patient files for guideline adherence. We also observed rearrangements of electronic patient records to facilitate guideline adherence, and some implemented electronic patient records if not already in use. Others showed schedules of regular patient file checks by the PQM or explained that they arranged additional in-service training, such as dealing with psychosocial factors. Also, some practices were already expanding the approach to other guidelines.

### Process evaluation

#### Fidelity

The programme’s emphasis for the individual PT was on the use of measurement instruments and the psychosocial factors, both identified by the practices as high-priority goals. Correct application of the ICF in the diagnostic process was not clearly present in the programme. All PTs went through the steps of self-regulation, although sometimes rather implicitly. Most self-assessments involved only general estimations of personal guideline adherence.

Contrary to the other tools to assist PQMs in their change management, attention to the management scan remained limited. There was ample opportunity for interaction in which the steps the managers made and problems they encountered were discussed and advice for implementation in the practice was provided. Advice about their leadership capacities was limited. The presence of both PTs and PQMs during four of six sessions created extensive interaction, enabling them to work together on their quality improvement plans and improving commitment.

Programme instructors competently delivered the methods and practical applications, albeit, due to time limitations, briefly for most. This resulted in some deficits in both programme delivery and learning, including superficial reflection by PTs on their personal adherence, limited attention for goal setting, management and leadership skills and little discussion of the issue of maintenance. Furthermore, changes in the programme were required because of the unexpected low knowledge levels of the PTs on some themes, such as red flags (signs and symptoms of serious diseases), application of measurement instruments and psychosocial factors. For that purpose, PTs advised a discussion about the content of the guideline in the first session.

#### Acceptability

The revised guideline was positively judged even though its recommendations were largely similar to the former version. The revision was unanimously found to be less normative, more flexible, less extensive and easier to understand and apply. The patient leaflet had only been used by one practice, despite the judgment of all practices that the content was supportive and useful.

Of the seven practices that completed the course, six were unanimously very positive about the programme (score 8 out of 10). In one practice, the opinions differed, varying from fair (6 out of 10) to very positive (8 out of 10). The main positive ideas were that it taught them something about the process of implementation of guidelines instead of treatment content and that it gave them the opportunity to compare themselves with other practices. The main critique came from one practice with a higher level management structure in that they had missed a ‘sparring partner’ on their own level. One practice dropped out. Although neither their pre-intervention adherence scores nor our observations showed better performance compared with other practices, managers explained the practice was already engaged in a quality improvement process and did not learn anything new. One of the practice’s two PQMs also indicated, however, to lack leadership skills, which may also have been a reason they dropped out. The other manager judged the programme as more suitable for practices with lower performance levels.

The PTs’ assessments of the course instructors were very positive, as were assessments of the interactive small group sessions with colleagues from the practice, the plenary discussions, presentations with peer and expert feedback, and the *Meet the Expert* session. Small group sessions with peers from other practices were highly appreciated by the managers, who learned from exchanging experiences, but to a lesser extent by individual PTs.

#### Feasibility

Problems with feasibility of the programme in its current form included available time, variability in completion of homework assignments, and underestimation of the needed remediation of the knowledge level for some issues. In addition, application of the self-regulation process (theoretical core) and sound clinical reasoning (basic professional skill) appeared to require more explicit instruction and guidance. Another participant’s advice was to more strictly monitor the translation to daily practice during the programme. Although all practices made progress, the plan for continuing the programme components as a normal part of practice would deserve greater attention and monitoring.

PQMs indicated that quality management in this form was valuable, yet time consuming, and that is would help if they were in some way financially compensated for their efforts. This was especially required when this time investment was expected to become part of daily routine. PTs realized that being involved in quality improvement activities would cost private time. Although they expressed commitment to do this, agreements had to be made with practice management about the required time investment. Concerning the number and duration of the sessions of the programme the PQMs and the PTs indicated that 3 hour-sessions were acceptable. Since the programme would benefit from an extra session to include the issues mentioned above, some participants preferred to make the individual sessions one hour longer instead. Moreover, a six-month, follow-up session would allow monitoring and support maintenance of the quality improvement process. Two PQMs indicated to prefer an on-site form of this programme, because it would reduce the time investment for the whole practice team. However, others indicated that this would reduce the opportunity for interaction and peer learning.

## Discussion

This pilot study evaluated the theory-based QUIP programme, aimed at enhancing PTs’ adherence to the Dutch guidelines for low back pain. For individual PTs, overall guideline adherence showed no improvement, but positive changes were observed for three individual quality indicators concerning the use of measurement instruments and handling psychosocial factors. These improvements were, however, associated with a large decrease in choosing correct patient profiles. For PQMs, the programme helped to structure practice quality management, provided tools to perform quality management, and supported the implementation of changes to improve quality of care for patients with low back pain. We also found favourable changes in motivational and affective determinants of guideline adherence at the individual performance level as well as in organizational determinants at the practice quality management level.

The absence of improvement in overall adherence was related to unexpected low knowledge levels of the PTs on some themes as well as to the short time span of the programme. Both factors hampered engagement of participants in all steps of the self-regulation process as the programme’s core strategy of change, and allowed detailed attention to only a limited number of important subjects. The improvements in individual quality indicators were associated with the multilevel approach of the QUIP programme, the formulation of individual performance goals and collective practice quality improvement goals, and PTs and PQMs collaborating to choose quality improvement strategies. In this respect, the substantial opportunity for interaction between PQMs and PTs appeared to be of vital importance.

A major strength of the study is its combined evaluation of potential effectiveness and process. This did not merely provide insight in the potency of the programme to improve guideline adherence, but also in the reasons for the presence or the absence in changes in adherence. A first limitation of the effect study is its one-group pre-test post-test design. The absence of a randomization procedure and a control group makes this design vulnerable to many forms of bias [36]. Although the findings of the process evaluation may be helpful in this respect, the rudimentary design implies that the potential effects of the programme should be interpreted with great caution. However, the main purpose of the effect evaluation was to assess whether further development of the programme would make sense at all. The results of this study indicate that the programme may have the capacity to improve guideline adherence. A second limitation concerns the many individual t-tests used to assess the various effects. This may result in an overestimation of effects due to alpha-inflation [44]. However, since our objective was to assess the potency of the programme to improve guideline adherence and adherence determinants, we preferred this more lenient alpha level in the spirit of discovery. Third, effect sizes to express the strength of the effects can be rather unstable in small samples, resulting in large confidence intervals [45]. As recommended for small samples, we therefore present them together with T-tests including significance levels [46]. A fourth limitation may be that guideline adherence was measured with a self-report measurement instrument, which may have caused overestimation [47,48]. Although standardized patients are considered the gold standard to measure practice performance [49], this method was considered far too expensive and time-consuming for our pilot study. Besides, the vignettes we applied had previously been found to have an acceptable level of validity [37,50-52]. A final limitation concerns the small, purposive and self-selected sample. Although age and gender of the individual participants did not substantially deviate from the national data [53], the results cannot be extrapolated to physical therapy practices that lack an initial quality management structure, or to PTs and PQMs that are less motivated for quality improvement. However, current developments in Dutch physical therapy, with health insurance companies monitoring the quality of care, will make an investment in the deliverance of optimal care inevitable. Hence, anticipating these developments in the quality certification of Dutch private practices physical therapy can be seen as a strength of our study.

Most published evaluations of interventions aimed at improving guideline adherence concern randomized clinical trials (RCTs), which makes a comparison of our study findings rather complicated. Mostly, improvements found in implementation research among health care providers, including physical therapy, are 5-15% [4,13-15]. Taking into account the limitations of our study, the 3% improvement in overall guideline adherence should be regarded as negligible and as being of no clinical importance. Improvements in individual quality indicators of 40% in the use of measurement instruments and of 24% in choosing treatment objectives including psychosocial factors may however be regarded as promising.

The improvements in individual quality indicators might be due to the fact that the QUIP programme focused on both the individual PT and the management levels. Such a multi-level approach has been recommended before [19,54], and found to be beneficial in improving the use of measurement instruments in physical therapy [55]. In our study, it provided positive experiences for PQMs and their PTs, enhancing commitment and showing that engaging in a quality improvement process together need not be burdensome or time consuming. Moreover, interaction between the levels within an organization is one of the core factors of organizational self-regulation [56]. This interaction may have enhanced feelings of peer and superior support, which have been recognized as important factors influencing guideline adherence [57].

As an adverse effect, we observed a decrease in the choice of the correct patient profile. Due to recent insights into the development of chronic pain, patient profiles in the revised low back pain guideline, which were used in our study, distinguish normal course low back pain from delayed course low back pain with absence or presence of psychosocial factors causing the delay. The guidelines recommend assessing psychosocial factors only for delayed course low back pain [22,23]. In our study, the attention given to psychosocial factors during the QUIP programme probably caused PTs to interpret the cases described in the vignettes predominantly as the profiles for which these factors are applicable, irrespective of the course of recovery. This is in line with recent research that emphasized the importance of early assessment and management of psychosocial factors because of their predictive value for chronic low back pain development [58-60]. This may indicate that the validity of profiles within the guideline is at stake and that psychosocial factors should, indeed, be assessed regardless of the course of recovery.

One explanation of the absence of an effect on overall guideline adherence may be that the time limitation of the programme forced PTs to prioritize, set goals and choose implementation strategies for a limited number of subjects. Although this collective goal setting is seen as a key factor in self-regulation and task performance [61], and revealed promising results on individual quality indicators, our findings indicate that PTs, as well as other health care professionals, may only be able to implement recommendations of guidelines one by one, or in small numbers simultaneously. This finding is in accordance with other guideline adherence or quality improvement studies [4,13,14,62-64]. As a consequence, complete guideline implementation might better be viewed as a stepwise process that requires an ongoing effort.

Participants judged the time investment of the programme as valuable but large. Some of them preferred an on-site intervention because they expected it to reduce their time investment. Although we have considered this approach, we chose for meetings outside the practice. Our choice for meetings with more than one practice simultaneously was mainly driven by four reasons. First, on-site interventions, such as educational outreach visits, have demonstrated small to modest effects [65] on the change of professional performance. Second, we wanted to create the opportunity for interaction, which is identified as a factor that may increase the effect of educational meetings [66]. Third, we preferred to take the PTs out of their daily context, since their habitual working environment provides cues for habitual performance [67]. By taking PTs out of their working environment these cues were avoided. Finally, we considered the costs of the programme, taking into account its implementation on a larger scale. Given the number of almost 5000 physical therapy practices in the Netherlands, an on-site programme would have been difficult to manage and very cost expensive.

Implementation of the programme on a larger scale requires trainers with knowledge and skills in physical therapy, education and management. In order to facilitate the dissemination we involved two trainers with the required knowledge and skills in physical therapy practice, physical therapy education and management education in the development of the programme. These trainers provide nationwide additional post qualifying education for physical therapists. The programme has now been included in their curriculum.

## Conclusion

In conclusion, although overall guideline adherence did not improve, changes on individual quality indicators suggest that a systematically developed, theory-based programme to enhance adherence to the Dutch physical therapy guidelines for low back pain has the potential to improve the quality of physical therapy care for patients with low back pain. The integrated approach of individual PTs and their PQMs, with much room for interaction, seems to benefit positive performance change. The self-regulation approach is suitable, but the programme should be re-designed in a way that allows for a thorough self-reflection on personal performance at the beginning and at the end. Also, the steps of self-regulation should be made more explicit to enhance PTs’ and PQMs’ awareness of this process. Goal setting, individually and collectively, and action planning seem to be core steps in the programme, but PTs and PQMs need some guidance in the sound formulation of challenging yet achievable goals for improvement. In addition, more attention should be paid to translation into daily practice and to the importance of the continuity of the self regulation process. Finally, the programme should result in a thorough future practice plan for guideline implementation. In order to achieve this, the programme should allow sufficient time for attention to individual subjects and strategies and could benefit from a follow-up session to assess and support sustainability. After re-designing the programme, more sophisticated designs with larger samples are required to draw sound conclusions about its effectiveness.

## Competing interests

The authors declare that they have no competing interests.

## Authors’ contributions

GR and JH conceived the study. All authors were involved in the development process of the intervention. GR was responsible for its implementation and for data collection and data analysis. AS was responsible for participant recruitment, data collection and data analysis. JH also participated in data analysis. GR drafted the manuscript, and JH, LKB, RABO and NKV contributed to manuscript review and revision. All authors read and approved the final manuscript.

## Pre-publication history

The pre-publication history for this paper can be accessed here:

http://www.biomedcentral.com/1472-6963/13/194/prepub

## Supplementary Material

Additional file 1 Synopsis of the QUIP programme. Click here for file

Additional file 2 Detailed overview of the Quality Improvement in Physical therapy (QUIP) programme. Click here for file
