# Supplementary material for: Evaluation of the theory-based Quality Improvement in Physical Therapy (QUIP) programme: a one-group, pre-test post-test pilot study
Source: BMC Health Serv Res. 2013 May 25;13:194. doi: 10.1186/1472-6963-13-194 (PMC3688482; doi:10.1186/1472-6963-13-194)
Supplement: Additional file 1 — Synopsis of the QUIP programme. [file 1472-6963-13-194-S1.doc]

**Additional file 1: Synopsis of the QUIP programme**

Since PTs and PQMs were expected to take responsibility for their own quality of care, self-regulation of individual and organizational learning was chosen as the main theoretical framework for the programme [1, 2]. The core steps of self-regulation are self-reflection, self-judgment, goal setting, planning and self-action [1, 2]. Self-regulation is an ongoing process during which various levels of an organization provide antecedents and reinforcements within and between levels.

The programme consisted of six meetings in a time period of three months: four 3-hour sessions for PTs and PQMs together and two 3-hour sessions for PQMs alone. The extensive opportunity for interaction between PTs and PQMs (who were also PTs) was expected to reinforce the quality improvement process [3]. Individual PTs assessed their personal adherence to the guidelines by comparing a patient record with the recommendations in the guidelines. Subsequently, each chose three points for individual improvement, which had to be specific, measurable, acceptable, realistic and time specific (SMART) [4]. Next, they were challenged to choose their own implementation strategy, to implement one of their objectives and to evaluate what had changed in their care process. Finally, they were asked to think about how they would maintain their actions. All this resulted in a personal development plan (PDP), that contained points for individual quality improvement, goals already achieved during the programme and unachieved goals, intended strategies, and a maintenance plan. Additionally, the PTs chose three collective goals with the colleagues and the PQMs from their practices.

Various methods and strategies were used to bring the PTs from self-reflection to their final evaluation and maintenance plan. We used self-monitoring by means of a reflection on a personal patient record. For information transfer, PTs attended brief lectures followed by deliberation and discussion and small group work. Vicarious learning and modeling was applied by a *Meet the Expert* session. We used guided practice in homework instruction, which was discussed and upon which participants received peer and expert feedback.

During two, extra separate sessions, PQMs were taught how to use a management scan (INK Quick Scan) [5] to assess improvement points for five organizational domains: leadership, strategy, means management, people management, and process management. The PQMs were also instructed on how to assess the organizational change culture using the Personal Change Style questionnaire [6] and how to perform a strengths, weaknesses, opportunities and threats (SWOT) analysis [7]. These instruments were chosen for practical convenience and ease of use rather than by scientific stringency. Additionally, PQMs were taught a strategy to select applicable change activities for their practice. They were challenged to make a risk assessment and a cost analysis for the change process. Finally, they planned how they would maintain their quality management. During the course, they were encouraged to find quick wins, goals that could be met in a short time and with relatively little effort. The process that was followed during the course resulted in a Practice Quality Improvement Plan (PQIP). This PQIP consisted of quality improvement goals, intended results, outcomes of the organizational analyses performed during the programme, chosen strategies, requirements, possible barriers and an expense estimate.

Since quality manager is a relatively new role in private practice physical therapy in the Netherlands, information transfer was used to a larger extent than for the individual PTs. There was much room for deliberation and discussion and small group work in which PQMs exchanged information and experiences. Guided practice comprised plenary presentation of plans and activities with discussion and peer and expert feedback.

Finally, the programme made use of the most current draft revision of the Dutch physical therapy guideline for low back pain (unpublished manuscript). The revised guideline was less comprehensive and was more comprehensible and user friendly to practitioners. Redundant text was removed to the separate explanation and justification document that accompanied the guideline. To explicitly support clinical reasoning, recommendations were directly linked to findings of a previous step in the process of care leading to recommendations such as “if you find...the guideline recommends to...”. As a means of additional support, PTs were provided with a patient information leaflet about guideline-adherent care as a means to manage patient expectations about physical therapy.

**References**

[1] Bandura, A. **Social Cognitive Theory of Self Regulation**. *Organ Behav Hum Decis Process* 1991;**50**:248-87.

[2] Boekaerts, M., Pintrich, P. R. and Zeidner, M., eds. **Handbook of Self Regulation**. San Diego: Elsevier Academic Press; 2005.

[3] Forsetlund, L., Bjorndal, A., Rashidian, A., Jamtvedt, G., O'Brien, M. A., Wolf, F., Davis, D., Odgaard-Jensen, J. and Oxman, A. D. **Continuing education meetings and workshops: effects on professional practice and health care outcomes**. *Cochrane Database Syst Rev* 2009(2):CD003030.

[4] Doran, G. **There's a S.M.A.R.T. way to write management's goals and objectives**. *Management review* 1981;**70**(19(AMA FORUM)):35-6.

[5] INK. **INK Quick Scan**. http://www.ink.nl/nl/p4d9c5f5f1a5fd/ink-quickscan.html. Accessed June 24, 2011.

[6] De Caluwe, L. and Vermaak, H. **Leren veranderen. Een handboek voor de veranderkundige**. Alphen a/d Rijn: Kluwer; 2006.

[7] Vermeylen, S. **Werken met de SWOT-analyse**. Brussel: Politeia; 2005.
