# Supplementary material for: Evaluation of the theory-based Quality Improvement in Physical Therapy (QUIP) programme: a one-group, pre-test post-test pilot study
Source: BMC Health Serv Res. 2013 May 25;13:194. doi: 10.1186/1472-6963-13-194 (PMC3688482; doi:10.1186/1472-6963-13-194)
Supplement: Additional file 2 — Detailed overview of the Quality Improvement in Physical therapy (QUIP) programme. [file 1472-6963-13-194-S2.doc]

**Additional file 2 Detailed overview of the QUality Improvement in Physical therapy (QUIP) programme**

| **POs / COs** | **Theoretical Methods and Practical applications** | **Actions** |
| --- | --- | --- |
| **session 1: general session with managers and PTs** | | |
| PT   - regular reflection on the content of work (self monitoring) - judging personal performance - enhance awareness of personal way of working - see the GL as a valuable quality tool - decide to make an effort to improve their adherence to the GL - keep patient records that contain sufficient information to enable reflection on the quality of their work   PQM   - POW / PQM decides to start a quality improvement project - POW/PQM plans and makes preparations for a quality improvement project | **Consciousness raising / Self reflection**   - Preparatory homework   **Knowledge transfer**   - Lectures - Individual Task   **Reflection on organization / Goal setting**   - Small group work with colleagues from practice   **Attitude building / Active learning**   - Plenary sessions with discussion and feedback | - Assessment of Personal way of Working by comparing a record of a patient with low back pain with the recommendations in the guidelines. Choose 5 items to improve. - Introduction to the objectives of the course. Brief introduction to the INK-management model and the Kleurenmodel van de Caluwé, which measures change culture, and introduction to the *SWOT analysis.* - What do the results of the Quick Scan-INK and the Personal Change Style questionnaire tell us. - Complete Personal Change Style questionnaire. - PQM and PTs select max 3 collective goals from their personal improvement goals - Perform a Quick Scan- INK (briefly introduced) of the practice which results in a view on the stage of development of the practice. - Organize the information of al assessments for the development of the Practice Quality Improvement plan. - Quality management and guidelines - positioning the guideline in quality management. - Deliberating the results of the group work and receive feedback and guidance. |
| **session 2a: managers** | | |
| PQM   - plans and makes preparations for a quality improvement project - manages the quality improvement project | **Knowledge transfer / Management Skills**   - Preparatory homework   **Consciousness raising / Organizational reflection / Knowledge transfer**   - Lectures / Plenary session   **Active learning / Guided practice**   - Plenary session with feedback/guidance/deliberation | - Start with steps 1-3 of the *Practice Quality Improvement plan*, and select three major issues for improvement. Consider 2 actions for quality improvement (Quick Wins) which you could perform in the next two weeks. Read the INK philosophy on the website. - Outcomes of the *Quick Scan-INK* are presented and discussed. Explanation of the underlying philosophy. - Explanation of assessments with the INK-model; the Kleurenmodel van de Caluwé, the SWOT-analysis, - Feedback on and recommendations for the temporary *Practice Quality Improvement plan*. Deliberation with PQMs of other practices. |
| **session 2b: general session with managers and PTs** | | |
| PT   - judge their personal performance - react on the basis of their judgement - correctly and completely assess the patients’ complaints in all the subsets of the ICF - categorize the patient correctly on the basis of duration of the episode, course and the presence of psychosocial variables (choose the correct patient profile) - choose adequate examination objectives and examination strategies - adequately apply questionnaires | **Goal setting / Skills training**   - Preparatory homework   **Implementation intentions / Skills training**   - Small group work with colleague from other practice   **Knowledge transfer / Active information processing / Organizational goal setting**   - Plenary session - Lecture - Deliberation / Discussion - Small group work with colleagues from practice | - Start with the first three steps of the *PDP.*  Select three out of five personal improvement goals and consider a plan for action. - Deliberate about and give feedback on each other’s *PDP* so far. Refine the three goals and make at least one of them S(pecific)M(easurable) A(cceptable) R(ealistic)T(ime specific). - Deliberation and feedback on the SMART goal – how SMART is it? - Diagnostics and use of questionnaires for patients with low back pain. - A case description (Profile 2) is used for deliberation and discussion about the diagnostics and the use of questionnaires. - How do we apply and interpret questionnaires we use in the practice. Set a SMART collective goal for the use of questionnaires in the practice. |
| **session 3a: managers** | | |
| PQM   - plans and makes preparations for a quality improvement project - manages the quality improvement project | **Management skills training**   - Preparatory homework   **Guided practice / Peer support**   - Plenary session with discussion and feedback   **Knowledge transfer**   - Lecture   **Active learning / Skills / Peer support**   - Small group work with PQM of other practice - Plenary session with feedback | - Write step 4-6 of the *PQIP* and review step 1-3. Makes changes if applicable. Be alert on possible Quick Wins. - Feedback on, deliberation with and suggestions of colleagues about the analysis in the *PQIP* - Strategies for quality improvement linked to the outcomes of the *Personal Change Style* questionnaire. Brief explanation of *Creative Problem Solving.* - Refine the analysis in the *PQIP* by means of deliberation and suggestions. - Discuss and find applicable strategies for your practice and prioritize them. Use the method of C*reative Problem Solving* for this purpose. - Presentation of the strategies and feedback |
| **session 3b: general session with managers and PT’s** | | |
| PT   - assesses psychosocial factors - integrates PS factors in the treatment-plan and decide about how to deal with these factors - addresses PS factors in the treatment of the patient with LBP - choose applicable treatment objectives and treatment strategies - apply the hands off approach in case of acute LBP with a normal course - apply a limited number of treatment sessions(max 4) in case of acute LBP with a normal course - provide adequate advice to the patient - formulate sound arguments when they deviate from the GLS recommendations   PQM   - manages the quality improvement project | **Self reflection / Skills training**   - Preparatory homework   **Knowledge transfer**   - Lecture   **Active learning**   - Plenary discussion   **Goal setting**   - Small work group with colleagues from practice (preceded by brief instruction)   **Modeling**   - Plenary session:Meet The Expert | - Complete step 5 of the *PDP* (process the feedback). Use the form for *Clinical reasoning* and apply it to one of your patients in practice were psychosocial factors are apparent. - PQM: Refine the collective objectives and make arrangements for implementation. - Treatment plan and treatment; dealing with psychosocial factors that impede recovery. - The prepared ‘cases’ are used for discussion about what a PT can do with psychosocial factors. - Deliberate about which factors are applicable for our practice and which are the most important factors? How do we deal with these factors and should this be changed? - Prepare questions for the *Meet the Expert-session.* - Demonstration, followed by an interactive session about how to diagnose and deal with psychosocial factors |
| **session 4: general session with managers and PTs** | | |
| PQM   - manages the quality improvement project - evaluates the quality improvement project and takes care of continuation   PT   - evaluate the effect of their action - maintain this procedure | **(Management) skills training**   - Preparatory homework   **Active learning / Peer support / Management skills training**   - PQM: Small work group with colleagues from other practice   **Organizational reflection / Monitoring**   - Plenary debate   **Active learning**   - PT individual task - Plenary debate - Plenary session | - PQM: Complete the PQIP - change strategies; risk analyses; cost calculation - PT: Complete the PDP – what did you change so far; did you reach your goal(s); how will you sustain in your quality improvement activities. - Deliberate about the various *PQIPs*. Is there anything you can use in another *PQIP* ? Discuss how you plan to sustain in your quality management. - Which changes in the practice have been made until now? How were they measured? How will the quality management be sustained? - Prepare a debate about your *PDP*. - Which results can be derived from the *PDPs*? - Closure: Evaluation and possible follow up arrangements |

PT = physical therapist; PQM = practice quality manager; GL = guideline

PO = performance objective; CO = change objective

PDP = personal development plan; PQIP = practice quality improvement plan
